# Effect of arsenic stress on 5-methylcytosine, photosynthetic parameters and nutrient content in arsenic hyperaccumulator *Pteris cretica* (L.) var. Albo-lineata

**DOI:** 10.1186/s12870-020-2325-6

**Published:** 2020-03-30

**Authors:** Veronika Zemanová, Marek Popov, Daniela Pavlíková, Pavel Kotrba, František Hnilička, Jana Česká, Milan Pavlík

**Affiliations:** 1 Isotope Laboratory, Institute of Experimental Botany, The Czech Academy of Sciences, Vídeňská 1083, 14220 Prague, Czech Republic; 2 Department of Agro-Environmental Chemistry and Plant Nutrition, Faculty of Agrobiology, Food and Natural Resources, Czech University of Life Sciences Prague, Kamýcká 129, 16500 Prague, Czech Republic; 3 Department of Biochemistry and Microbiology, University of Chemistry and Technology, Technická 5, 16628 Prague, Czech Republic; 4 Department of Botany and Plant Physiology, Faculty of Agrobiology, Food and Natural Resources, Czech University of Life Sciences Prague, Kamýcká 129, 16500 Prague, Czech Republic

**Keywords:** *Pteridaceae*, Long-term stress, Toxic element, Epigenetic change, DNA demethylation

## Abstract

**Background:**

Arsenic toxicity induces a range of metabolic responses in plants, including DNA methylation. The focus of this paper was on the relationship between As-induced stress and plant senescence in the hyperaccumulator *Pteris cretica* var. Albo-lineata (*Pc*-Al). We assume difference in physiological parameters and level of DNA methylation in young and old fronds as symptoms of As toxicity.

**Results:**

The As accumulation of *Pc*-Al fronds, grown in pots of haplic chernozem contaminated with 100 mg As kg^− 1^ for 122 days, decreased with age. Content of As was higher in young than old fronds for variants with 100 mg As kg^− 1^ (2800 and 2000 mg As kg^− 1^ dry matter, respectively). The highest As content was determined in old fronds of *Pc*-Al grown in pots with 250 mg As kg^− 1^. The increase with age was confirmed for determined nutrients – Cu, Mg, Mn, S and Zn. A significant elevation of all analysed nutrients was showed in old fronds. Arsenic accumulation affected DNA methylation status in fronds, but content of 5-methylcytosine (5mC) decreased only in old fronds of *Pc*-Al (from 25 to 12%). Determined photosynthetic processes showed a decrease of fluorescence, photosynthetic rate and chlorophylls of As treatments in young and old fronds. Water potential was decreased by As in both fronds. Thinning of the sclerenchymatous inner cortex and a reduction in average tracheid metaxylem in the vascular cylinder was showed in roots of As treatment. Irrespective to fronds age, physiological parameters positively correlated with a 5mC while negatively with direct As toxicity. Opposite results were found for contents of Cu, Mg, Mn, S and Zn.

**Conclusions:**

The results of this paper point to changes in the metabolism of the hyperaccumulator plant *Pc*-Al*,* upon low and high exposure to As contamination. The significant impact of As on DNA methylation was found in old fronds. Irrespective to fronds age, significant correlations were confirmed for 5mC and As toxicity. Our analysis of the very low water potential values and lignification of cell walls in roots showed that transports of assimilated metabolites and water between roots and fronds were reduced. As was showed by our results, epigenetic changes could affect studied parameters of the As hyperaccumulator plant *Pc*-Al, especially in old fronds.

## Background

Environmental pollution with arsenic (As) poses a risk to plant, animal and human health. Uptake and accumulation of this element by plants vary according to plant species. For most plants, significantly reduced growth and fitness is evident at soil arsenic concentrations of 25.0–85.0 mg kg^− 1^ total As [1]. In contrast, some fern species of the *Pteridaceae* family can tolerate As and accumulate it in their above-ground tissues to > 1000 mg As kg^− 1^ dry weight [2, 3]. The cultivars of *Pteris cretica* (var. Albo-lineata, Wimsetti and Alexandrae) were identified as an arsenic hyperaccumulators by Zhao et al. [3], who reported that this species accumulates As to the levels found in *P. vittata*, the first As-hyperaccumulating species identified. Arsenate (As^V^) taken up by roots of *P. vittata* from the soil was reduced to arsenite (As^III^), which was rapidly transported to the vacuoles of the upper and lower epidermal cells and trichomes of the fronds [4]. Tu and Ma [5] found that As in the fronds of *P. vittata* was primarily contained as inorganic arsenite (average of 94%). According to these authors arsenite re-oxidation to arsenate occurred more often with senescence of fronds. Koller et al. [6] confirmed the effect of the development stage of fronds of *Pteris umbrosa* on As content. The senesced fronds had a significantly lower As content in contrast to green fronds while expanding fronds had the highest As content. The different result - increase in As concentration from young to mature and old fronds was shown in *P. vittata* [5].

Arsenic stress can provoke numerous toxic effects in plants. As is widely reported to inhibit the rate of photosynthesis in plants and to reduce chlorophyll concentration [7]. Agnihotri and Seth [8] showed a decline in photosynthetic pigments and gaseous exchange parameters in plants exposed to As, indicating the onset of senescence. Foyer and Noctor [9] also confirmed that oxidative stress activates senescence associated with the degradation of photosynthetic pigments and remobilises the basic nutrients C, N, P, and S. Modified stress metabolism due to extended reversible senescence releases nutrients from catabolic processes and transports them from old leaves into young leaves more efficiently.

Studies have also revealed that stress-inducing abiotic factors, including As, can trigger epigenetic changes (in particular DNA methylation/demethylation), which may contribute to the regulation of gene expression in chronic stress conditions [10]. Phenotypic manifestations of epigenetic changes include reduced plant growth (dwarfism) and development (in particular seed germination [11]), flowering period [12, 13] and male fertility/sterility of anthers and/or pollens [14].

The primary detoxification of As in the cells of terrestrial plants relies on rapid reduction of As^V^ to As^III^ and the formation of As^III^-glutathione or As^III^-phytochelatin complexes, which are eventually transported to the vacuole. Disturbances in cellular processes, caused by a toxic excess of As, induce oxidative stress responses [15–17], methylation of both As forms in *P. cretica* [18], and epigenetic changes in DNA [19]. In this study, we aimed to gain insight into the context of As hyperaccumulation and plant senescence in *Pteris cretica* var. Albo-lineata. In addition, we examined related changes in selected physiological parameters and DNA methylation status as potentially indicative of epigenetic modification. The degree of senescence was evaluated with respect to different changes found in young and old fronds of *P. cretica* var. Albo-lineata.

## Results

### Growth and elemental content of As-exposed *P. cretica* var. Albo-lineata

**Fig. 1:**
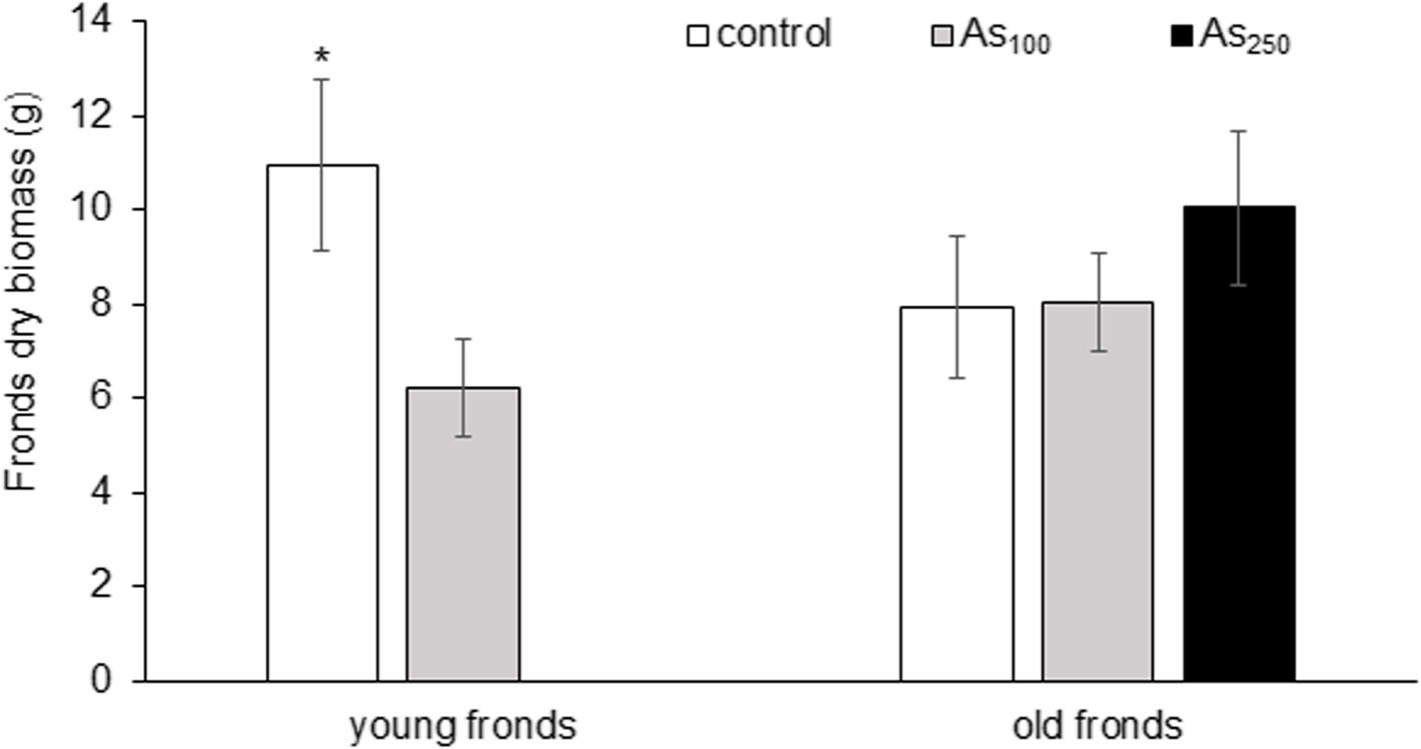
Dry biomass of young and old fronds of *P. cretica* var. Albo-lineata. Values represent mean ± SD. The Kruskal-Wallis test was used to compare the significance (*p* < 0.05) between: i) treatments of young fronds (control and As_100_) and old fronds (control, As_100_ and As_250_) and ii) young and old fronds of control and As_100_ treatment. Treatments significantly different from each other are marked with asterisks. Differences between young and old frond was not significant

The effect of As_100_ soil was observed only on young fronds. The dry biomass of *Pc*-Al young fronds was decreased by 43% (Fig. 1). The effect of As_250_ soil was not observed. Differences between young and old fronds of *Pc*-Al were not statistically significant. Symptoms of As toxicity were not observed.

**Fig. 2:**
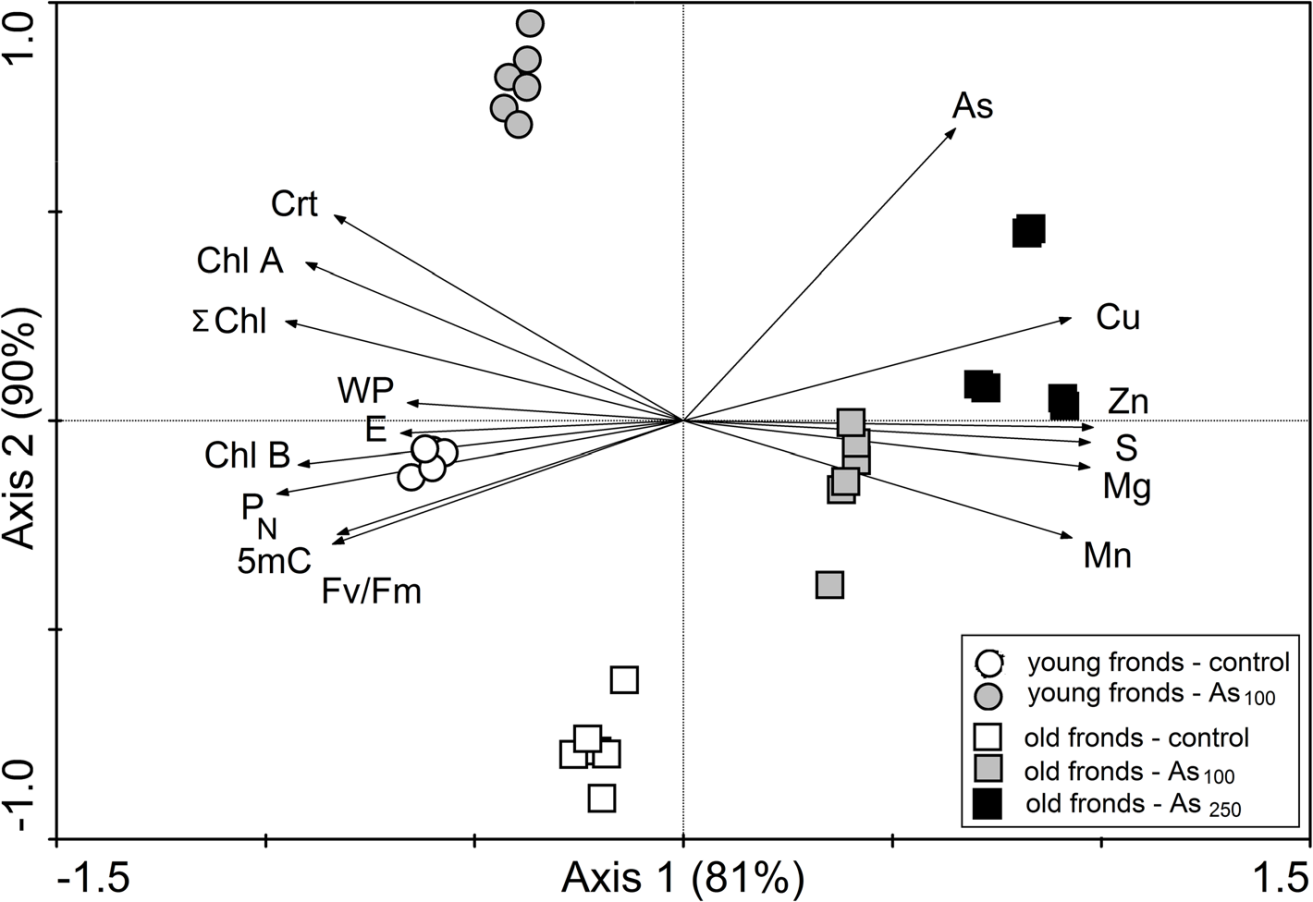
Ordination diagram showing the results of PCA analysis with selected parameters in fronds of *P. cretica* var. Albo-lineata. Treatment abbreviations: control, treated with 0 mg As kg^− 1^ soil; As_100_, treated with 100 mg As kg^− 1^ soil; As_250_, treated with 250 mg As kg^− 1^ soil. Parameter abbreviations: Crt, carotenoids; Chl A, chlorophyll a; Chl B, chlorophyll b; Σ Chl, total chlorophyll; WP, water potential; P_N_, net photosynthetic rate; E, transpiration rate; Fv/Fm, fluorescence; 5mC, 5-methylcytosine; As, Cu, Mg, Mn, S and Zn; total content of elements

The analysis of the concentration of elements in dried fronds of *Pc*-Al revealed that the highest As concentration was determined in As_250_ old fronds (Table 1). In the control and As_100_ soil condition, young fronds had approximately 1.5 times higher concentrations of As than old ones (Table 1). Compared with controls, ferns grown in the As_100_ soils increased As concentrations 150- and 170-fold in young and old fronds, respectively. Ferns grown in the As_250_ soil increased As concentration 421-fold compared to control. Irrespective of the presence of added As in the soils, old fronds tended to accumulate higher concentrations of Cu, Mg, Mn, S and Zn than young fronds (Table 1, Fig. 2). The effect of high soil As – As_100_ and As_250_ on the Cu accumulation showed an increase in young fronds (by 17%) and old fronds (by 100%), respectively. The increases in S (59%) and Zn (86%) concentration were only observed in old fronds. When grown in As_100_ and As_250_ soils, the concentrations of Mg and Mn increased in old fronds of *Pc*-Al (Mg by 60% and Mn by 66%) but decreased in young fronds (Mg by 6% and Mn by 18%).

**Table 1:** Content of elements in young and old fronds of *P. cretica* var. Albo-lineata

Parameters	Young fronds (mg kg^− 1^ dry weight)		Old fronds (mg kg^− 1^ dry weight)		
	control	As_100_	control	As_100_	As_250_
	x̄ ± SD	x̄ ± SD	x̄ ± SD	x̄ ± SD	x̄ ± SD
As	19 ± 0.6^aB^	2847 ± 63^bB^	12 ± 0.1^aA^	2034 ± 47^abA^	5056 ± 143^b^
Cu	5.1 ± 0.1^aA^	6.0 ± 0.1^bA^	5.5 ± 0.1^aB^	7.0 ± 0.2^abB^	11 ± 0.2^b^
Mg	2277 ± 61^bA^	2139 ± 43^aA^	2509 ± 40^aB^	3342 ± 35^abB^	4034 ± 106^b^
Mn	28 ± 0.5^bA^	23 ± 0.9^aA^	35 ± 2^aB^	52 ± 1.3^abB^	58 ± 4^b^
S	1326 ± 27^aA^	1372 ± 43^aA^	1521 ± 51^aB^	2195 ± 17^abB^	2420 ± 179^b^
Zn	17 ± 0.2^aA^	18 ± 0.7^aA^	21 ± 0.4^aB^	27 ± 0.3^abB^	39 ± 1.2^b^

Values with the same letter were not statistically significant at the 0.01 level by the Kruskal-Wallis test. Different letters indicate significantly different values (*p* < 0.01): a, b comparison between the treatments of young fronds (control and As_100_) and old fronds (control, As_100_ and As_250_); A, B comparison between young and old fronds for control and As_100_ treatment. Results for comparison between control and As_100_ of young and old fronds are in Additional File 1. Coefficients of variation (CV, %) are in Additional File 2.

### DNA methylation status of As-exposed *P. cretica* var. Albo-lineata

**Fig. 3:**
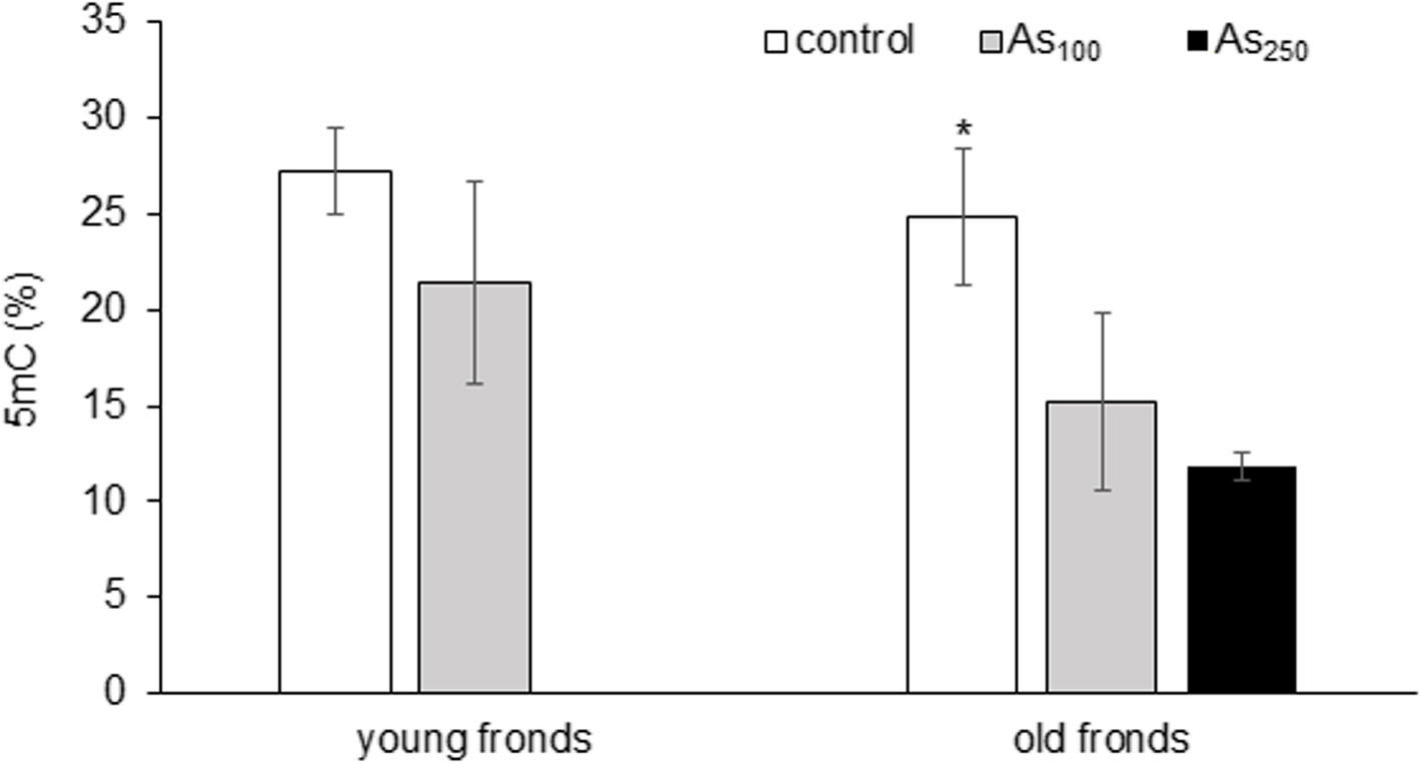
Content of 5-methylcytosine (5mC) in young and old fronds of *P. cretica* var. Albo-lineata. Values represent mean ± SD. The Kruskal-Wallis test was used to compare the significance (*p* < 0.05) between: i) treatments of young fronds (control and As_100_) and old fronds (control, As_100_ and As_250_) and ii) young and old fronds of control and As_100_ treatment. Treatments significantly different from each other are marked with asterisks. Differences between young and old frond was not significant. Coefficients of variation (CV, %) are in Additional File 2

Since As and senescence might affect the methylation of DNA at cytosines in plants, the 5-methylcytosine content (5mC, %) of *Pc*-Al DNA was analysed (Figs. 2 and 3). Compared with controls, the overall DNA methylation status in fronds of ferns grown in As_100_ soils was fluctuated from 27 to 21% in young and from 25 to 15% in old fronds. The 5mC content in old fronds of ferns grown in As_250_ soil was 12%. The decrease was only proved in old fronds. The effect of frond senescence on 5mC content was not statistically significant, but the trend, in terms of average 5mC content, was lower in old fronds.

### Pigment content, fluorescence, WP and GEP of As-exposed *P. cretica* var. Albo-lineata

Growth in As_100_ and As_250_ soil resulted in a decrease of chlorophyll contents (Chl A, Chl B and Σ Chl) of *Pc*-Al (Table 2). The content of carotenoids (Crt) was reduced by As, but not significantly. Irrespective of the presence of added As in the soil, contents of all analysed pigments were higher in young fronds than in old fronds. Compared with controls, pigment content of ferns grown in the As_100_ soils was higher in young fronds, especially Chl A and Crt contents (5-fold and 6-fold higher than those in old fronds, respectively). While the average value of Chl A and Chl B ratio remained unaffected by As in old fronds, it increased in young fronds of ferns grown in As_100_ soils (Table 2).

**Table 2:** Physiological parameters in young and old fronds of *P. cretica* var. Albo-lineata

Parameters	Young fronds		Old fronds		
	control	As_100_	control	As_100_	As_250_
	x̄ ± SD	x̄ ± SD	x̄ ± SD	x̄ ± SD	x̄ ± SD
Chl A (nmol ml^−1^)	11 ± 0.2^bB^	10 ± 0.5^aB^	2.6 ± 0.6^bA^	1.5 ± 0.4^abA^	1.0 ± 0.5^a^
Chl B (nmol ml^− 1^)	4.8 ± 1.0^bB^	2.8 ± 0.2^aB^	2.6 ± 0.7^bA^	1.9 ± 0.4^abA^	0.6 ± 0.2^a^
Chl A/Chl B (−)	2.4 ± 0.4^aB^	3.6 ± 0.03^bB^	1.1 ± 0.7^aA^	0.9 ± 0.5^aA^	1.4 ± 0.4^a^
Σ Chl (nmol ml^− 1^)	16 ± 1.0^bB^	13 ± 0.7^aB^	5.2 ± 0.1^bA^	3.4 ± 0.3^abA^	1.6 ± 0.7^a^
Crt (nmol ml^− 1^)	2.4 ± 0.1^aB^	2.3 ± 0.3^aB^	0.5 ± 0.2^aA^	0.3 ± 0.1^aA^	0.3 ± 0.04^a^
Fv/Fm (µmol m^-2^ s^-1^)	0.8 ± 0.02^bB^	0.7 ± 0.03^aB^	0.7 ± 0.02^bA^	0.6 ± 0.07^aA^	0.5 ± 0.1^a^
WP (MPa)	−1.4 ± 0.09^aB^	−1.9 ± 0.03^bB^	−1.7 ± 0.04^aA^	−3.7 ± 0.03^bA^	−2.5 ± 0.4^ab^
E(mmol H_2_O m^−2^ s^−1^)	0.7 ± 0.2^aA^	0.9 ± 0.2^bA^	0.8 ± 0.1^bA^	0.7 ± 0.1^aA^	0.3 ± 0.04^a^
P_N_ (μmol CO_2_ m^− 2^ s^− 1^)	8.1 ± 0.1^bB^	7.7 ± 0.04^aB^	7.7 ± 0.04^bA^	7.2 ± 0.03^abA^	6.7 ± 0.1^a^
WUE (−)	12 ± 2.7^bA^	8.6 ± 1.3^aA^	9.7 ± 1.2^aA^	9.4 ± 0.6^aA^	24.4 ± 1.6^b^

Chlorophyll fluorescence (Fv/Fm), as an indicator of plant photosynthetic activity, was lower in old fronds than in young fronds. The value of Fv/Fm for young fronds from control plants (0.82 μmol m^− 2^ s^− 1^) responded to the quantum yield of photosystem II. During As_100_ stress Fv/Fm of fronds decreased compared with controls (by 12.5% in young fronds and 14% in old fronds). Also As_250_ stress decreased Fv/Fm of old fronds – by 29% (Table 2). The lowest value of Fv/Fm (71% of the value observed with the controls) was measured in As_250_ conditions in old fronds. Observed declines in pigment content and Fv/Fm in old leaves of fern in As_100_ and As_250_ soils indicated a faster progression of senescence.

**Fig. 4:**
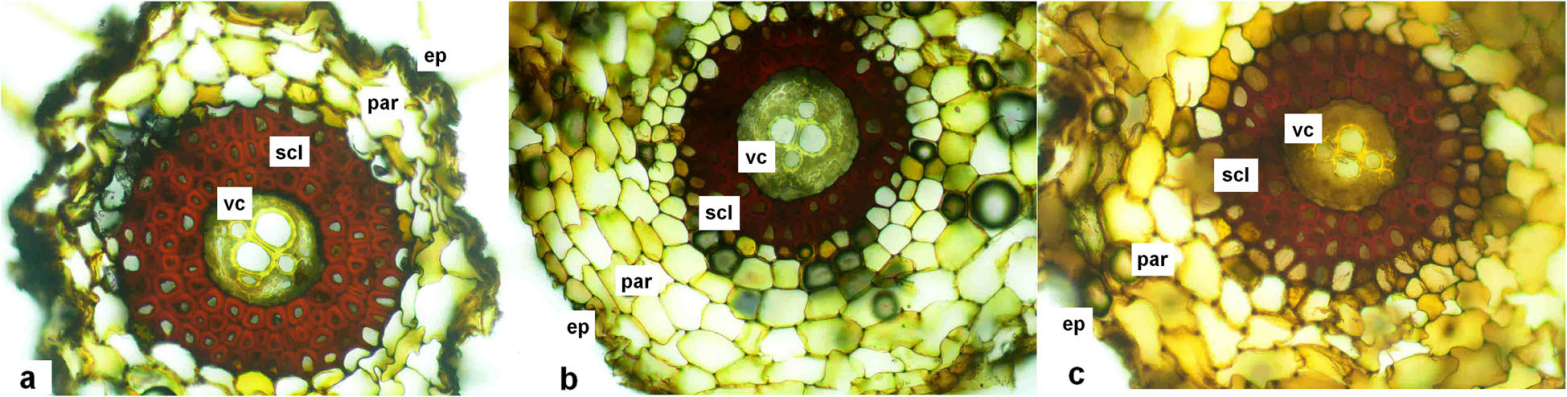
Cross-section through an adventitious root of *P. cretica* var. Albo-lineata. Treatment: control (4a; 0 mg As kg^− 1^ soil); As_100_ (4b; 100 mg As kg^− 1^ soil); As_250_ (4c; 250 mg As kg^− 1^ soil). Abbreviations: vc, vascular cylinder; scl, sclerenchymatous inner cortex; par, parenchymatous outer cortex; ep, epidermis

Water potential (WP) was decreased in young and old fronds of the plants grown in As_100_ soil (by 36 and 118%, respectively). Higher values of WP were observed in young fronds, irrespective of added As. To explore potential changes in roots, cross-section analysis through adventitious roots was performed. The roots of As treated plants showed thinning of the sclerenchymatous inner cortex and a reduction in average tracheid metaxylem in the vascular cylinder, compared to controls (Fig. 4).

From these data, the water-use efficiency was estimated (WUE = P_N_/E). Values with the same letter were not statistically significant at the 0.01 level by the Kruskal-Wallis test. Different letters indicate significantly different values (*p* < 0.01): a, b comparison between the treatments of young fronds (control and As_100_) and old fronds (control, As_100_ and As_250_); A, B comparison between young and old fronds for control and As_100_ treatment. Results for comparison between control and As_100_ of young and old fronds are in Additional File 1. Coefficients of variation (CV, %) are in Additional File 2.

The P_N_ and the rate of transpiration (E) were determined to gain further insight into the photosynthetic performance in *Pc*-Al fronds (Table 2). The P_N_ and E data indicated a higher photosynthetic activity in the young fronds of the control ferns (Table 2, Fig. 2), and a decrease in the photosynthesis rate under As conditions (by 5% in young fronds and 13% in old fronds). 28.5% increase of transpiration was observed in young fronds after As application. Compared with controls, added As decreased WUE only in young fronds (by 29%).

### Principal component analysis of physiological parameters

The first axis of the PCA analysis explained 81% of the variability of all analysed data, the first two axes explained 90% of the variability, and the first four axes together explained 99% of the variability. Diagramming PCA analysis was used for visualisation of all relationships between *Pc*-Al parameters (Fig. 2; data only for As_100_ are in Additional File 3). In the PCA diagram, the first ordination axis divided the young fronds group on the left side from old fronds on the right side. This division indicated a large effect of frond senescence on all studied parameters. For young and old fronds, marks for treatments (control, As_100_) were located in the different parts of the diagram, which indicated a high effect of the treatments on all the recorded data. As observed with primary data, PCA confirmed that the accumulation of Cu, S, Zn, Mg and Mn was more pronounced in old fronds of *Pc*-Al grown in As_100_ and As_250_ soils and that Chl B, P_N_, WP, E, Fv/Fm, as well as 5mC were higher in young fronds of control plants. Arsenic content was negatively correlated with relative 5mC content of DNA as the angle between the vectors for As and 5mC was > 90°. Relationships visualised in the PCA diagram were confirmed by linear correlations (Table 3). The results in Table 3 showed an effect of As and 5mC of all treatments on other measured parameters. Correlations of As effect and 5mC on other parameters were calculated in different old fronds, where senescence was evaluated as a difference of tested parameters between young and old fronds (Table 3). The content of As in *Pc*-Al fronds significantly correlated with 5mC and other physiological parameters, except Crt. Negative relationships of As were confirmed for pigments, Fv/Fm, WP, E and P_N_. By comparison, these parameters were positively correlated with 5mC. Negative relationships were found between 5mC and Cu, Mg, Mn, S and Zn. By comparison, these parameters were positively correlated with As. The effect of As and 5mC of low As treatment (As_100_) is showed in Additional File 4.

**Table 3:** Linear correlation of As and 5mC with selected parameters of *P. cretica* var. Albo-lineata

As	r	5mC	r
5mC	−0.74^***^	As	−0.74^***^
Mg	0.71^***^	Mg	−0.77^***^
Cu	0.89^***^	Cu	−0.76^***^
Zn	0.79^***^	Zn	−0.76^***^
Mn	0.59^**^	Mn	−0.74^***^
S	0.69^***^	S	−0.78^***^
Chl A	−0.38^*^	Chl A	0.59^**^
Chl B	−0.75^***^	Chl B	0.69^***^
Crt	−0.31^n.s.^	Crt	0.56^**^
Fv/Fm	−0.77^***^	Fv/Fm	0.74^***^
WP	−0.41^*^	WP	0.64^***^
E	−0.56^***^	E	0.44^*^
P_N_	−0.87^***^	P_N_	0.81^***^

^*^p < 0.05, ^**^p < 0.01, ^***^p < 0.001; ^n.s.^, not statistically significant

## Discussion

Our results show that when grown in chernozem soils spiked with 100 and 250 mg As kg^−1^, As could accumulate in the fronds of *P. cretica* var. Albo-lineata to > 2000 mg As kg^− 1^ dry mass. These data confirmed the As hyperaccumulation status of *Pc*-Al and were consistent with results reported by Zhao et al. [3] for this fern and by Tu and Ma [5] for *P. vittata*. The As content in young fronds of control and As_100_ variants was higher than in old fronds. Effect of the development stage of fronds on As content was determined in *Pteris umbrosa* by Koller et al. [6]. During maturation of young fronds and senescence, As concentration in *P. umbrosa* exposed to 100 and 600 mg As L^− 1^ declined. This finding is consistent with our results for control and As_100_ variants.

Arsenic stress induces epigenetic changes in organisms, resulting in a decrease or increase in DNA methylation [19]. Analysis of 5mC in *Pc*-Al showed that As reduced the extent of DNA methylation. Similar results for heavy metals were published by Aina et al. [20]. The first paper focused on the effect of As on DNA methylation in plants was published by Erturk et al. [21]. Their results showed DNA hypermethylation of some genes in germinating maize seeds exposed to low As levels.

An increase of DNA methylation increases plant growth and transcriptionally represses genes involved in flavonoid biosynthesis [22]. A decrease of DNA methylation reduces plant growth and stimulates flowering, formation and growth of buds [13, 23, 24]. This finding was confirmed by our results. Dry biomass of *Pc*-Al young fronds was decreased by 43% (Fig. 1). As revealed by PCA analysis (Fig. 2), physiological parameters of the plant are affected more strongly by the methylation status of *Pc*-Al DNA than by direct As toxicity.

Some publications suggest that parts of DNA are sensitive to epigenetic changes [25]. However, in plants, the conservative parts of DNA without changes in DNA methylation were observed. Little information about the epigenetic activation of transcription of silenced plant genes of primary and secondary metabolites is known. Cazzonelli [26] described epigenetic changes linked to the regulation of metabolic pathways leading to carotenoid biosynthesis in relation to abscisic acid (control of carotenogenesis). According to Zhang et al. [27] epigenetic changes are linked with the biosynthesis of chlorophylls and tocopherols whose precursor is phytyl diphosphate. Lushchak and Semchuk [28] were also interested in these epigenetic changes. According to these authors, plants can increase photosynthesis by chlorophylls biosynthesis or by synthesizing the antioxidant metabolites tocopherols. We showed the continuity of changes in methylation/demethylation of cytosine DNA in relation to the photosynthetic pigments carotenoids and chlorophylls (primary relationship) and also to gas-exchange parameters (GEP) or to Fv/Fm, which are indicators of plant photosynthetic activity.

It has been well documented that stress-related senescence processes involve the degradation of photosynthetic pigments [9], accompanied by a reduction in photosynthetic efficiency. Our results indicated that excess As reduced the level of chlorophylls and affected photosynthetic processes in *Pc*-Al fronds (Fig. 2, Tables 2 and 3). Farooq et al. [29] reported that As decreased GEP and pigment content in *Brassica napus* and, according to Wang et al. [30] this toxic element significantly affected Fv/Fm in *P. vittata* within 60 days of exposure. An association between epigenetic changes in old fronds, resulting from As stress, and a reduced chlorophyll content might be indicated by the correlation between 5mC and Chl A and Chl B levels (Table 3). The decline in the levels of carotenoids and chlorophylls in young fronds of As_100_ treated plants was less than that seen in older fronds. A significant increase of the Chl A/Chl B ratio was confirmed for young fronds of As_100_ plants. These changes, together with the values of P_N_, E and Fv/Fm, pointed to a senescence in As_100_ plants. During senescence, plant metabolites are remobilised from old leaves to young leaves after expression of genes typical for senescence [31]. We found that As toxicity slightly increased leaf senescence (Fig. 2, Tables 1, 2 and 3). The opposite correlations results for 5mC, in contrast to direct As toxicity (Table 3), showed that decreases of chlorophylls, fluorescence and P_N_ could be affected by epigenetic changes. Ay et al. [32] published similar conclusions for the effects of epigenetic changes on physiological processes in plants.

A decrease of 5mC content in As plants led to a significant negative correlation with increased amounts of Cu, Zn and Mn, cofactors of superoxide dismutases, and with S, a key element in the biosynthesis of cysteine and methionine. Increased accumulation of tested elements could be affected by epigenetic changes as showed by the significant correlations for 5mC (Table 3). While there was an elevation in the concentrations of Cu, Mn, and Zn, in *P. vittata* exposed to As contamination [5], in *Pc*-Al it was more pronounced in old fronds as compared to young ones*.* These authors observed a same trend as we did – higher Mg content in old fronds compared to young ones in *P. vittata*. It was reasonable to assume that these elements were significant as cofactors of antioxidative metalloenzymes [33–35] as a part of chlorophylls (Mg), and as non-enzymatic antioxidants, protecting against As-induced stress. Increased concentrations of S can be linked to the accumulation of glutathione and phytochelatins involved in detoxification of cellular As in *P. cretica* var. Mayii [36]. Based on this, the observation that the concentration of S and Zn remained unaffected and the concentrations of Mn and Mg were reduced in young fronds of *Pc*-Al grown in As_100_ soil was surprising. We hypothesised that changes in Mn and Mg content were linked with N metabolism.

Water consumption by ferns is directly proportional to As contamination. One of the causes of changes in water content in fronds is the lignification of the conducting tissues in the roots. We found very low WP values for As_100_ and As_250_ plants as a result of stress attributable to As contamination (Table 2). Induced stress in cell walls leads primarily to the lack of water in fronds and secondarily to osmotic stress, which is a limiting factor for the growth and development of these plants. Leaf senescence together with the effect of As resulted in lignification of conducting tissue (Fig. 4). Similar reports of WP reduction as a result of the lignification of conducting tissue in plants exposed to stress conditions were published by Hare and Cress [37] and Yamaguchi et al. [38]. If the photosynthetic membrane system is protected by flavonoids, ascorbate and tocopherols [28, 39–41], then cell walls are protected by lignin [38]. Reduced DNA methylation in *Pc*-Al increases biosynthesis of sterols, tocopherols, flavonoids, isoflavonoids and lignins because these plant metabolites are epigenetically regulated by silencing genes [40, 42, 43]. Cross-sections through adventitious roots of As_100_ and As_250_ plants showed the deformation of root cell walls as a result of lignification (Fig. 4). Zanella et al. [44] observed morphological changes in tobacco roots growing in As and Cd contaminated solutions. They found increased cell wall thickness due to lignin over-deposition in the rhizodermal and external cortical parenchyma cells of the primary structure zone, which led to premature exodermis formation. Similar changes in root, lignification upon exposure to metals were confirmed by Piršelová et al. [45]. According to cited works, the lignification-induced cellular changes result in a reduction in water uptake by plants. This finding is in line with our results: changes in WP values and morphological changes in the roots. Reduction of water content and metabolites subsequently limits the ability of the plant to overcome As toxicity.

## Conclusions

The results of this paper point to changes in the metabolism of the As-hyperaccumulator plant *Pteris cretica* var. Albo-lineata*,* on exposure to 100 and 250 mg As kg^− 1^ contamination. Compared with controls, ferns grown in the As_100_ soils increased As concentrations in young and old fronds, 150- and 170-fold, respectively. Ferns grown in the As_250_ soil increased As concentration 421-fold compared to control. Higher As content was found in the young fronds in comparison to old fronds of control and As_100_ treatments. Analysis of 5mC content showed that accumulation of As was associated with affected DNA methylation. The decrease of 5mC was confirmed in old fronds (from 25 to 15% and 12% in contrast to control). As revealed by PCA and correlations, physiological parameters of the *Pteris cretica* var. Albo-lineata are strongly affected by the methylation status of DNA and by direct As toxicity. Increased accumulation of tested nutrients (Cu, Mn, Zn, Mg and S) or decreased chlorophylls, Fv/Fm, P_N_ and WP could be affected by epigenetic changes as was showed by our results.

Accumulation of As in plants affected photosynthetic processes and the content of pigments in fronds. There was a decrease of chlorophylls (ΣChl by 69%) and Fv/Fm (by 29%) in old fronds of As treatment, indicating a faster progression of senescence. These changes together with values of P_N_ and E, indicated reversible senescence in As plants. Based on our determination of very low values for WP (from − 1.4 to − 3.7) and morphological changes in the roots (lignification of cell walls) we proposed that transport of assimilated metabolites between roots and fronds might be reduced.

## Methods

### Plant material and experimental design

Plants of *Pteris cretica* (L.) var. Albo-lineata (*Pc*-Al) were obtained from the garden centre Tulipa Praha (Czech Republic). Ferns at the 10–15 fronds stage were planted in 5 L pots (1 fern for pot) under greenhouse conditions (natural photoperiod; temperature 22–24 °C; relative humidity approximately 60%) for 122 days. Each pot contained 5 kg of haplic chernozem mixed with 0.5 g N, 0.16 g P and 0.4 g K per 1 kg of soil (supplemented as NH_4_NO_3_ and K_2_HPO_4_). The soil used in this experiment (Table 4) was collected from a non-polluted area in Prague-Suchdol, Czech Republic (50°8ˊ8˝ N, 14°22ˊ43˝ E). Ferns were grown in this soil without As supplement (control) and with two As dose - 100 mg As per kg soil (As_100_) and 250 mg As per kg soil (As_250_). Arsenic was added as a solution of Na_2_HAsO_4_ and was thoroughly mixed with the soil; maturation period of spiked soil was ten days. Each treatment was replicated three times. Above-ground biomass of control and As_100_ variants were separated to young and old fronds. The young and old fronds were separated according to their location in plant habit and their size. The young fronds were located lower ground of fern and their area did not exceed to 5 × 10 cm. The larger fronds from full habit were indicated as old fronds. Growth of new fronds were not found at As_250_ variants and senescent fronds were indicate as old fronds. After being harvested, the fronds were treated as described below. Cross-sections through an adventitious root were inspected using a Nikon E 200 microscope equipped with DS camera head and the NIS-Elements application (Nikon Instruments, Inc., Melville, NY, USA).

**Table 4:** Basic properties of soil

pH_KCl_	C_org_	CEC	Total As	Water extractable As	Sand	Silt	Clay	Bulk density
(−)	(%)	(mmol_+_ kg^−1^)	(mg kg^− 1^)	(mg kg^− 1^)	(%)	(%)	(%)	(g cm^− 3^)
7.1	1.83	258	16 ± 1.7	0.10 ± 0.01	26	72	2	2.57

C_org_, organic carbon; CEC, cation exchange capacity.

### Determination of arsenic and other elements

Fronds were oven-dried for three days at 40 °C. Homogenised material (0.5 ± 0.05 g) was digested with a mixture of HNO_3_ and H_2_O_2_ (4:1, v/v) in an Ethos 1 device (MLS GmbH, Leutkirch im Allgäu, Germany). Contents of As, Cu, Mg, Mn, and Zn were determined using inductively coupled plasma-optical emission spectrometry (ICP-OES; Agilent 720, Agilent Technologies Inc., Santa Clara, CA, USA). Certified reference material (CRM NIST 1573a Tomato leaves, Analytika®, Czech Republic) was mineralized under the same conditions for quality assurance.

### Isolation of DNA and determination of relative DNA methylation status based on % 5-methylcytosine

The fronds were weighed, frozen in liquid nitrogen and stored at − 80 °C prior to DNA methylation analysis. To isolate total DNA, the fronds (1 g fresh weight) were ground to a fine powder in liquid nitrogen by mortar and pestle. DNA was extracted from 100 mg of powdered tissue using a NucleoSpin Plant II molecular kit (Macherey-Nagel GmbH & Co. KG, Düren, Germany), as instructed in the user manual. The global DNA methylation status of DNA was determined using 100 ng of isolated DNA and a MethylFlash Methylated DNA Quantification Kit (Fluorometric; Epigentek Group Inc., Farmingdale, NY, USA) according to the manufacturer’s instructions. A SpectraMax MiniMax 300 Imaging Cytometer (Molecular Devices LLC, San Jose, CA, USA) with excitation at 530 nm was used to measure the fluorescence at 590 nm.

### Determination of pigments

Pigment content in the leaves was measured photometrically with an Evolution 2000 UV-Vis (Thermo Fisher Scientific Inc., Waltham, MA, USA). A vessel-free leaf segment (0.5 cm^2^) excised from a freshly separated frond was incubated in the dark in 1 ml dimethylformamide with shaking for 24 h. The absorbance of the extract was measured at wavelengths 480, 646.8, and 663.8 nm. Absorbance values at 710 nm were subtracted from these measurements. Data pigment contents were calculated from these data:

Chlorophyll A (Chl A; nmol ml^− 1^): Chl A = 12.0 × A_663.8_–3.11 × A_646.8_.

Chlorophyll B: (Chl B; nmol ml^− 1^): Chl B = 20.78 × A_646.8_–4.88 × A_663.8_.

Total chlorophyll (Σ Chl; nmol ml^− 1^): Chl A + Chl B = 7.12 × A_663.8_ + 17.67 × A_646.8_.

Carotenoids (Crt; nmol ml^− 1^): Crt_x + c_ = (1000 × A_480_–1.12 Chl A – 34.07 Chl B) / 245.

### Determination of fluorescence

The chlorophyll fluorescence [variable fluorescence (Fv)/maximal fluorescence (Fm); μmol m^− 2^ s^− 1^] was measured using a modulated chlorophyll fluorometer OS1-FL (Opti-Sciences, ADC, BioScientific, Ltd., Hoddesdon, UK). The fresh leaf was obscured by clipping after 20 min to set up a dark-adapted state. Chlorophyll fluorescence was excited by a 660 nm solid-state light source, with filters blocking radiation longer than 690 nm. Saturation of the photosystem being measured was achieved by using a filtered 35 W halogen lamp (350–690 nm) with a pulse of 15,000 μmol m^− 2^ s^− 1^ during 0.8 s.

### Determination of water potential

Water potential (WP; MPa), a measure of the energy status of the water in a system, was measured using a dew point PotentiaMeter (Decagon Devices, Inc., Pullman, WA, USA). The leaves of the plants were placed in a disposable syringe, the air was drawn off from the syringe, and the syringe was tightly closed with parafilm. The specimen was frozen at − 18 °C, then thawed, and the sap flow was pushed out into the measuring chamber of the PotentiaMeter.

### Determination of selected photosynthesis parameters with gas-exchange parameters (GEP)

The net photosynthetic rate (P_N_; μmol CO_2_ m^−2^ s^−1^) and the rate of transpiration (E; mmol H_2_O m^−2^ s^−1^) were determined with the portable gas exchange system LCpro+ (ADC BioScientific, Ltd., Hoddesdon, UK). The water-use efficiency parameter (WUE) was calculated from these determined values (WUE = P_N_/E). Values of P_N_ and E were determined between 8:00 and 11:30 Central European Time (CET) and conditions of measurement chamber were described previously [46, 47].

### Statistical analysis

All data were checked for homogeneity of variance and normality by Levene and Shapiro-Wilk tests. Collected data did not meet the conditions for the use of analysis of variance (ANOVA) and were thus evaluated by non-parametric Kruskal-Wallis test in the Statistica 12.0 program (StatSoft, Inc., Tulsa, OK, USA). Significant differences were assessed as effect of i) treatment on physiological parameters and ii) age of fronds on physiological parameters. A principal component analysis (PCA), in the CANOCO 4.5 program, was applied to all collected data as a single set. We used standardisation of species because data of different characters were analysed together. PCA was used to draw correlations from the complex data set. The results were visualised in the form of bi-plot ordination diagrams using the CanoDraw program [48]. Correlations were confirmed using a linear correlation (r, *p* < 0.05, *p* < 0.01, *p* < 0.001) by Statistica 12.0.

## Supplementary information

**Additional file 1. Table S1.** Content of elements and physiological parameters in young and old fronds of *P. cretica* var. Albo-lineata growing on low As dose – As_100_.

**Additional file 2. Table S2.** Coefficients of variation (CV, %) for the content of elements, DNA methylation and physiological parameters in *P. cretica* var. Albo-lineata.

**Additional file 3. Figure S1.** Ordination diagram showing the results of PCA analysis with selected parameters in fronds of *P. cretica* var. Albo-lineata growing on low As dose – As_100._ Treatment abbreviations: control, treated with 0 mg As kg^− 1^ soil; As_100_, treated with 100 mg As kg^− 1^ soil. Parameter abbreviations: Crt, carotenoids; Chl A, chlorophyll a; Chl B, chlorophyll b; Σ Chl, total chlorophyll; WP, water potential; P_N_, net photosynthetic rate; E, transpiration rate; Fv/Fm, fluorescence; 5mC, 5-methylcytosine; As, Cu, Mg, Mn, S and Zn; total content of elements. The first axis of the PCA analysis explained 74% of the variability of all analysed data, the first two axes explained 94% of the variability, and the first four axes together explained 99% of the variability.

**Additional file 4. Table S3.** Linear correlation of As and 5mC with selected parameters of *P. cretica* var. Albo-lineata growing on low As dose – As_100_.

## Data Availability

The datasets used and/or analysed during the current study available from the corresponding author on reasonable request. The plant material was bought from the Tulipa Praha garden centre (Czech Republic). No other permissions were necessary to buy and to cultivate these plants.

## Abbreviations

5mC: 5-methylcytosine content
As_100_: Treatment with 100 mg As per kg soil
As_250_: Treatment with 250 mg As per kg soil
Chl A: Chlorophyll A
Chl B: Chlorophyll B
Crt: Carotenoids
E: Rate of transpiration
Fv/Fm: Chlorophyll fluorescence
GEP: Gas-exchange parameters
PCA: Principal Component Analysis
*Pc*-Al: *Pteris cretica* var. Albo-lineata
P_N_: Net photosynthetic rate
WP: Water potential
WUE: Water-use efficiency
