# Supplementary material for: Effect of arsenic stress on 5-methylcytosine, photosynthetic parameters and nutrient content in arsenic hyperaccumulator Pteris cretica (L.) var. Albo-lineata
Source: BMC Plant Biol. 2020 Mar 30;20:130. doi: 10.1186/s12870-020-2325-6 (PMC7106808; doi:10.1186/s12870-020-2325-6)
Supplement: Supplementary file 1 — Additional file 1. Table S1. Content of elements and physiological parameters in young and old fronds of P. cretica var. Albo-lineata growing on low As dose – As100. [file 12870_2020_2325_MOESM1_ESM.docx]

Additional file 1: Table S1 Content of elements and physiological parameters in young and old fronds of *P. cretica* var. Albo-lineata growing on low As dose – As_100_.

| Parameters | Young fronds |  | |  | | Old fronds | |  |
| --- | --- | --- | --- | --- | --- | --- | --- | --- |
|  | (mg kg^-1^ dry weight) | | |  | | (mg kg^-1^ dry weight) | | |
|  | control | As_100_ | |  | | control | | As_100_ |
|  | x̄ ± SD | x̄ ± SD | |  | | x̄ ± SD | | x̄ ± SD |
| As | 19 ± 0.6^aB^ | 2847 ± 63^bB^ |  | | 12 ± 0.1^aA^ | | 2034 ± 47^bA^ | |
| Cu | 5.1 ± 0.1^aA^ | 6.0 ± 0.1^bA^ |  | | 5.5 ± 0.1^aB^ | | 7.0 ± 0.2^bB^ | |
| Mg | 2277 ± 61^bA^ | 2139 ± 43^aA^ |  | | 2509 ± 40^aB^ | | 3342 ± 35^bB^ | |
| Mn | 28 ± 0.5^bA^ | 23 ± 0.9^aA^ |  | | 35 ± 2^aB^ | | 52 ± 1.3^bB^ | |
| S | 1326 ± 27^aA^ | 1372 ± 43^aA^ |  | | 1521 ± 51^aB^ | | 2195 ± 17^bB^ | |
| Zn | 17 ± 0.2^aA^ | 18 ± 0.7^aA^ |  | | 21 ± 0.4^aB^ | | 27 ± 0.3^bB^ | |
| Chl A (nmol ml^-1^) | 11 ± 0.2^bB^ | 10 ± 0.5^aB^ |  | | 2.6 ± 0.6^bA^ | | | 1.5 ± 0.4^aA^ |
| Chl B (nmol ml^-1^) | 4.8 ± 1.0^bB^ | 2.8 ± 0.2^aB^ |  | | 2.6 ± 0.7^bA^ | | | 1.9 ± 0.4^aA^ |
| Chl A/Chl B (-) | 2.4 ± 0.4^aB^ | 3.6 ± 0.03^bB^ |  | | 1.1 ± 0.7^aA^ | | | 0.9 ± 0.5^aA^ |
| Σ Chl (nmol ml^-1^) | 16 ± 1.0^bB^ | 13 ± 0.7^aB^ |  | | 5.2 ± 0.1^bA^ | | | 3.4 ± 0.3^aA^ |
| Crt (nmol ml^-1^) | 2.4 ± 0.1^aB^ | 2.3 ± 0.3^aB^ |  | | 0.5 ± 0.2^aA^ | | | 0.3 ± 0.1^aA^ |
| Fv/Fm (µmol m^-2^ s^-1^) | 0.8 ± 0.02^bB^ | 0.7 ± 0.03^aB^ |  | | 0.7 ± 0.02^bA^ | | | 0.6 ± 0.07^aA^ |
| WP (MPa) | -1.4 ± 0.09^aB^ | -1.9 ± 0.03^bB^ |  | | -1.7 ± 0.04^aA^ | | | -3.7 ± 0.03^bA^ |
| E (mmol H_2_O m^-2^ s^-1^) | 0.7 ± 0.2^aA^ | 0.9 ± 0.2^bA^ |  | | 0.8 ± 0.1^aA^ | | | 0.7 ± 0.1^aA^ |
| P_N_ (µmol CO_2_ m^-2^ s^-1^) | 8.1 ± 0.1^bB^ | 7.7 ± 0.04^aB^ |  | | 7.7 ± 0.04^bA^ | | | 7.2 ± 0.03^aA^ |
| WUE (-) | 12 ± 2.7^bA^ | 8.6 ± 1.3^aA^ |  | | 9.7 ± 1.2^aA^ | | | 9.4 ± 0.6^aA^ |

From these data, the water-use efficiency was estimated (WUE = P_N_/E). Values with the same letter were not statistically significant at the 0.01 level by the Kruskal-Wallis test. Different letters indicate significantly different values (p < 0.01): a, b comparison between the treatments of young and old fronds (control and As_100_); A, B comparison between young and old fronds for control and As_100_ treatment.
