# Supplementary material for: Effect of arsenic stress on 5-methylcytosine, photosynthetic parameters and nutrient content in arsenic hyperaccumulator Pteris cretica (L.) var. Albo-lineata
Source: BMC Plant Biol. 2020 Mar 30;20:130. doi: 10.1186/s12870-020-2325-6 (PMC7106808; doi:10.1186/s12870-020-2325-6)
Supplement: Supplementary file 2 — Additional file 2. Table S2. Coefficients of variation (CV, %) for the content of elements, DNA methylation and physiological parameters in P. cretica var. Albo-lineata. [file 12870_2020_2325_MOESM2_ESM.docx]

Additional file 2: Table S2 Coefficients of variation (CV, %) for content of elements, DNA methylation and physiological parameters in *P. cretica* var. Albo-lineata.

| CV (%) of parameters | Young fronds |  |  | Old fronds |  |  |
| --- | --- | --- | --- | --- | --- | --- |
|  | control | As_100_ |  | control | As_100_ | As_250_ |
| As | 3 | 2 |  | 1 | 2 | 3 |
| Cu | 2 | 1 |  | 2 | 4 | 2 |
| Mg | 3 | 2 |  | 2 | 1 | 3 |
| Mn | 2 | 4 |  | 6 | 3 | 6 |
| S | 2 | 3 |  | 3 | 0.8 | 7 |
| Zn | 0.9 | 4 |  | 2 | 1 | 3 |
| 5mC | 10 | 30 |  | 18 | 37 | 6 |
| P_N_ | 0.6 | 0.5 |  | 0.5 | 0.4 | 2 |
| WUE | 22 | 15 |  | 12 | 6 | 7 |
| Chl A | 2 | 5 |  | 24 | 25 | 52 |
| Chl B | 20 | 6 |  | 27 | 23 | 36 |
| Chl A/Chl B | 15 | 1 |  | 60 | 52 | 27 |
| Σ Chl | 6 | 5 |  | 3 | 8 | 46 |
| Crt | 4 | 11 |  | 36 | 29 | 40 |
| Fv/Fm | 2 | 4 |  | 3 | 13 | 30 |
| WP | 7 | 2 |  | 3 | 0.8 | 14 |
| E | 30 | 24 |  | 17 | 18 | 11 |
