# Supplementary figures and images for: Effect of arsenic stress on 5-methylcytosine, photosynthetic parameters and nutrient content in arsenic hyperaccumulator Pteris cretica (L.) var. Albo-lineata
Source: BMC Plant Biol. 2020 Mar 30;20:130. doi: 10.1186/s12870-020-2325-6 (PMC7106808; doi:10.1186/s12870-020-2325-6)

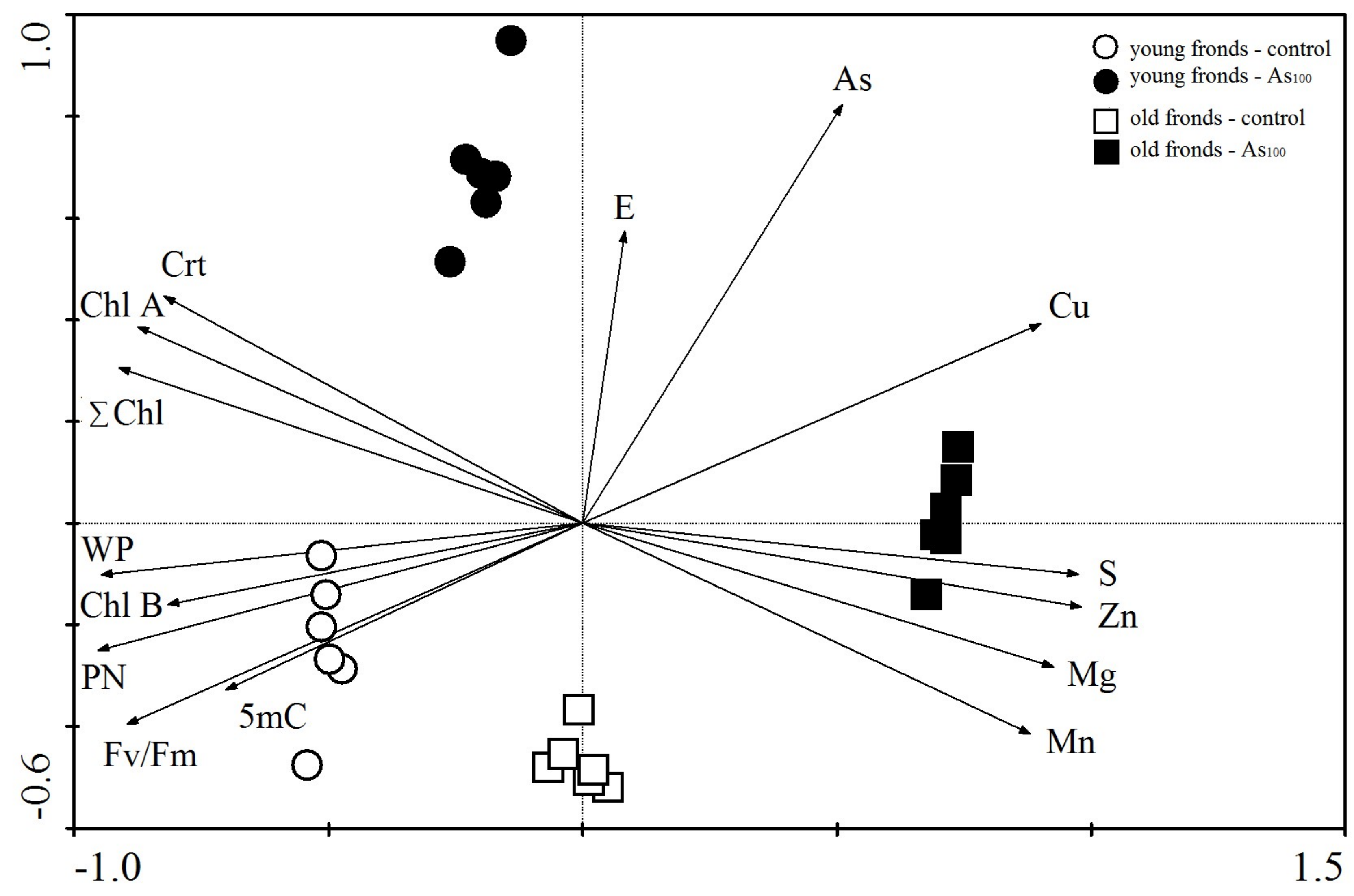

Supplement: Supplementary file 3 — Additional file 3. Figure S1. Ordination diagram showing the results of PCA analysis with selected parameters in fronds of P. cretica var. Albo-lineata growing on low As dose – As100. Treatment abbreviations: control, treated with 0 mg As kg− 1 soil; As100, treated with 100 mg As kg− 1 soil. Parameter abbreviations: Crt, carotenoids; Chl A, chlorophyll a; Chl B, chlorophyll b; Σ Chl, total chlorophyll; WP, water potential; PN, net photosynthetic rate; E, transpiration rate; Fv/Fm, fluorescence; 5mC, 5-methylcytosine; As, Cu, Mg, Mn, S and Zn; total content of elements. The first axis of the PCA analysis explained 74% of the variability of all analysed data, the first two axes explained 94% of the variability, and the first four axes together explained 99% of the variability. [file 12870_2020_2325_MOESM3_ESM.pdf]
