# Supplementary material for: Effect of arsenic stress on 5-methylcytosine, photosynthetic parameters and nutrient content in arsenic hyperaccumulator Pteris cretica (L.) var. Albo-lineata
Source: BMC Plant Biol. 2020 Mar 30;20:130. doi: 10.1186/s12870-020-2325-6 (PMC7106808; doi:10.1186/s12870-020-2325-6)
Supplement: Supplementary file 4 — Additional file 4. Table S3. Linear correlation of As and 5mC with selected parameters of P. cretica var. Albo-lineata growing on low As dose – As100. [file 12870_2020_2325_MOESM4_ESM.docx]

Additional file 3: Table S3 Linear correlation of As and 5mC with selected parameters of *P. cretica* var. Albo-lineata growing on low As dose – As_100_.

| As | r |  | 5mC | r |
| --- | --- | --- | --- | --- |
| 5mC | -0.54^**^ |  | As | -0.54^**^ |
| Mg | 0.15^n.s.^ |  | Mg | -0.59^**^ |
| Cu | 0.69^***^ |  | Cu | -0.74^***^ |
| Zn | 0.25^n.s.^ |  | Zn | -0.63^***^ |
| Mn | 0.05^n.s.^ |  | Mn | -0.55^**^ |
| S | 0.31^n.s.^ |  | S | -0.66^***^ |
| Chl A | 0.02^n.s.^ |  | Chl A | 0.43^*^ |
| Chl B | -0.49^*^ |  | Chl B | 0.48^*^ |
| Fv/Fm | -0.74^***^ |  | Fv/Fm | 0.72^***^ |
| WP | -0.50^*^ |  | WP | 0.70^***^ |
| E | 0.43^*^ |  | E | -0.18^n.s.^ |
| P_N_ | -0.62^***^ |  | P_N_ | 0.70^***^ |

^*^ p < 0.05, ^**^ p < 0.01, ^***^ p < 0.001; ^n.s.^, not statistically significant.
